# In Situ Observation of Bubbles and the Effect of Ultrasonic Vibration on Bubble Behavior in EDM

**DOI:** 10.3390/ma16206676

**Published:** 2023-10-13

**Authors:** Chenxue Wang, Tomohiro Sasaki, Atsutoshi Hirao

**Affiliations:** 1 Graduate School of Science and Technology, Niigata University, Niigata 950-2181, Japan; f21k007f@mail.cc.niigata-u.ac.jp; 2 Faculty of Engineering, Niigata University, Niigata 950-2181, Japan; tomodx@eng.niigata-u.ac.jp; 3 Faculty of Education, Niigata University, Niigata 950-2181, Japan

**Keywords:** USV assisted EDM, bubble behavior, CFD

## Abstract

Accumulation and concentration of debris in deep hole electrical discharge machining (EDM) significantly hinder its machining efficiency and accuracy. It is believed that the movement of bubbles associated with the discharge gap flow field play a pivotal role in debris removal and influence the discharge conditions. Ultrasonic vibration (USV) of the electrode is thought to be an effective method for improving EDM-generated bubbles and debris exclusion. In this study, we first elucidated the behavior of bubbles during EDM of holes with varying aspect ratios. Subsequently, USV was introduced to EDM. The behavior of dielectric fluid flow under the influence of ultrasonic vibration was analyzed using computational fluid dynamics (CFD), which revealed time-varying changes in discharge gap flow pressure and velocity. The velocity of the dielectric flow field near the electrode’s side face was found to reach a maximum of approximately 15.2 m/s, greatly facilitating debris removal. High-speed camera observations revealed that bubbles were dispersed within the side gap, with most of them adhering to the electrode’s wall. Furthermore, the bubbles exhibited a tendency to continuously break up and coalesce near the hole’s outlet before escaping in the USV-assisted EDM. These observed characteristics of bubble behavior under the influence of USV are expected to significantly enhance debris removal and promote efficient dielectric exchange.

## 1. Introduction

Electrical discharge machining (EDM) technology is a formidable asset in advancing manufacturing toward miniaturization and precision [1]. Its unique processing characteristics, which do not depend on the physical hardness of materials or involve contact force, have generated significant interest in researching and applying EDM within mainstream manufacturing, particularly in precision machining. Nevertheless, there are some limitations in the field, notably in EDM drilling of micro holes and EDM sinking for deep molds, where machining depth and aspect ratio remain constrained to a certain extent.

In the realm of electrical discharge machining (EDM), the breakdown discharge process is accompanied by several intricate phenomena. These include the vaporization of both the electrode material and working fluid, the decomposition of molecules, and the ionization of atoms. During the moment of breakdown, a substantial volume of high-pressure gaseous material is generated. This leads to a rapid expansion of the interface between the high-pressure gaseous material and the working fluid, occurring at a rate of several tens of meters per second. As the discharge concludes, ions and electricity recombine, giving rise to neutral particles while the vaporized atoms and molecules solidify into process remnants or working liquids. Simultaneously, gases such as oxygen and methane are trapped, eventually forming bubbles [2]. These bubbles, along with machining debris, grow and accumulate within the discharge gap. Consequently, this issue impedes the circulation of dielectric fluid and the removal of machining debris, thereby restricting both the aspect ratio of holes and machining efficiency [3]. Moreover, in the context of micro-EDM, targeting smaller and higher-aspect-ratio micro holes can lead to operational interruptions due to excessive tool wear [4]. Jin Wang et al. verified that bubble expansion significantly contributes to debris exclusion from the bottom gap. They achieved this through meticulous observations of debris and bubble movement, employing a finely designed experimental apparatus [5]. This confirmation underscores the vital role played by bubble behavior in facilitating debris removal and enabling efficient dielectric exchange.

**Figure 1:**
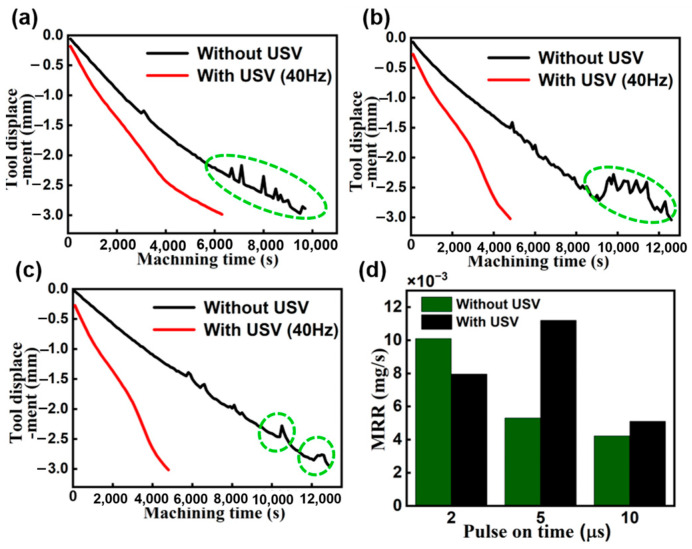
Electrode displacement in different machining pulse duration time: (**a**) 2 μs, (**b**) 5 μs, and (**c**) 10 μs. (**d**) The effect of ultrasonic vibration on MRR with different pulse duration times.

Until now, much of the research in this area has been centered on the fabrication of micro holes with high aspect ratios using EDM. This body of work has delved into various methods and numerical calculations aimed at enhancing debris removal and improving dielectric exchange [6]. Tomohiko et al. investigated the machining characteristics of micro-EDM using ultrasonic vibration in machining fluid under ultra-low discharge energy conditions. Their findings indicated significant improvements in machining speed and a reduction in lateral gap width when ultrasonic vibration was applied to fluids in micro-EDM [7]. Similarly, we investigated the influence of ultrasonic vibration on the EDM performance. We conducted a quantitative analysis and comparison between conventional EDM and USV-assisted EDM. The diameter of 3 mm of copper rod and SKD 61 tool steel were adopted as the electrode and workpiece, respectively. The ultrasonic amplitude and vibration frequency at the electrode tip were 2 μm and 40 kHz, respectively. As depicted in Figure 1. The machining speed gradually decreased with increasing feeding depth in the EDM without ultrasonic vibration. The micro tool exhibited fluctuations toward the end of machining, indicated by the green dotted line in Figure 1a–c. It is worth noting that this phenomenon may be attributed to debris, and there was no evidence of bubble accumulation when ultrasonic vibration was applied. Due to frequent instances of abnormal discharge and short circuits, the tool retreated frequently during further machining in EDM without assistance from USV. From an examination of the impact of USV on the material removal rate (MRR), as shown in Figure 1d, it is apparent that application of ultrasound vibration can improve the MRR, particularly in EDM under the high-energy condition. Moreover, USV was applied to the EDM of micro holes with a large depth-to-diameter ratio on titanium alloy by Qixuan Xing et al. [8]. They concluded that the MRR increased by 2.4 times, the RTWR (relative tool wear rate) decreased by 65.8%, θ (taper angle) reduced by 73%, and OC (overcut) decreased by 32% at the ultrasonic amplitude of 6 μm. Amir Abdullah et al. studied the effect of ultrasonic-assisted EDM on the surface integrity of cemented tungsten carbide (WC-Co). They reported that the tool ultrasonic vibration of electrode significantly improves the surface integrity of cemented tungsten carbide (WC-Co10%) during EDM [9]. Additional studies have focused on developing specialized electrode structures and modifying dielectric fluid-flushing methods. These efforts have led to notable enhancements in the deteriorating interelectrode environment during micro-EDM, consequently increasing machining efficiency [10,11,12]. Although extensive research has been presented on improving the machining characteristics of ultrasonic vibration-assisted EDM from an experimental perspective. Limited attention has been devoted to elucidating the micromechanics governing debris exclusion and bubble behavior in ultrasonic vibration-assisted EDM. With the advancement of high-speed imaging technology, Kunieda pioneered the use of transparent electrodes for observing gap phenomena; however, this was primarily restricted to SiC single crystals and did not incorporate ultrasonic vibration [13]. In comparison to that of traditional EDM, observing bubble behavior and debris exclusion in ultrasonic vibration-assisted EDM presents challenges due to facility limitations, cavitation, and the complexity of bubble dynamics. Consequently, alternative approaches, such as numerical analysis of flow fields and bubble dynamic simulations, have been proposed. Mohammad T. Shervain-Tabar et al. conducted a series of numerical studies on the hydrodynamic behavior of dielectric fluids and bubble dynamics. They revealed the evolution of vapor bubbles generated by electrical discharges and their interactions with the surrounding fluid using the boundary integral equation method. Their findings also suggested that tool vibration significantly increases material removal rates in USV-assisted EDM when the frequencies of electrical discharge and tool vibration are synchronized [14,15,16]. However, due to the absence of bubble behavior observations and experimental demonstrations of debris motion, most studies have overlooked the coexistence of bubbles and debris. Furthermore, in USV-assisted EDM, the critical importance of observing bubble movement lies in its role in optimizing process parameters, improving material removal efficiency, enhancing surface quality, and managing electrode wear. Understanding the dynamics of bubbles during machining is essential for fine-tuning the process and achieving optimal results [5].

In this paper, we aim to present an in-depth analysis of bubble behavior and debris exclusion from the discharge gap. We focus on the growth and coalescence of bubbles and explore bubble behavior in response to the fluctuating pressure field induced by ultrasonic vibration of the tool electrode. This paper is structured as follows: Section 2 details the system configuration and in situ observations of bubble behaviors in EDM. Section 3 and Section 4 present the results of CFD simulations concerning interelectrode fluid flow and bubble behavior in USV-assisted EDM of high-aspect-ratio holes. Finally, Section 5 summarizes our findings and provides conclusions.

## 2. In Situ Observations of Bubble Behavior in EDM

### 2.1. Experimental Method and Observation Equipment

**Figure 2:**
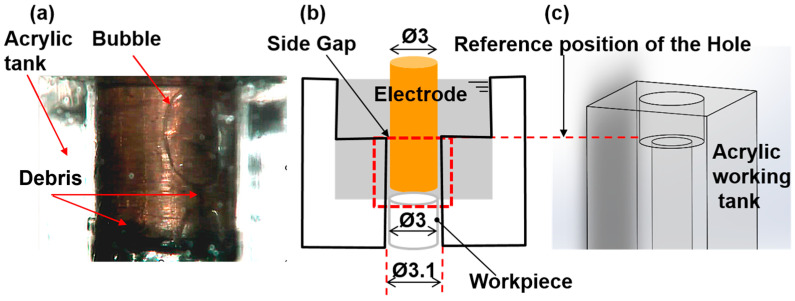
Schematic of the experimental method to realize in situ observation: (**a**) the observed shape of bubbles and debris, (**b**) the schematic diagram of the bubble observation device, (**c**) three-dimensional model diagram.

**Figure 3:**
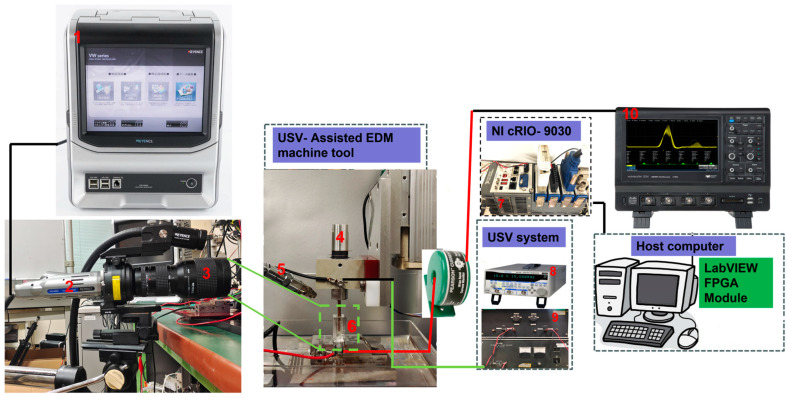
The schematic diagram of the experimental apparatus for the in situ bubble observation system: 1—VW-9000 HIGH SPEED MICROSCOPE; 2—VW—6000 C HIGH SPEED lens (color); 3—MACRO ZOOM SYSTEM VW—Z2; 4—Bolt-clamped Langevin type transducer (HEC-1540P2BF); 5—Pump nozzle; 6—Observation area; 7—CompactRIO Controller cRIO 9030; 8—FUNCTION GENERATOR SG-4105; 9—WIDEBAND POWER AMPLIFIER; 10—Wavesurfer 3034 oscilloscope.

Observing the gap area is challenging for many workpieces, as they are not transparent. Therefore, as illustrated in Figure 2, we present an observation device specifically designed for studying bubble behavior. We employ a square acrylic tank with a side length of 17.5 mm for this purpose, and its walls are covered with reflective tinfoil, except for the surface designated for observation. To simulate the discharge gap, a through-hole with a diameter of 3.1 mm is precisely drilled at the center of the working tank. A workpiece with a diameter of 3.0 mm is inserted from the bottom end into the working tank, and the gap is sealed with super glue. The upper surface of the 3.1 mm diameter hole serves as the reference point for measuring the hole depth, which is defined as the depth at which the electrode is inserted. The side gap width is maintained at 0.05 mm to align with the actual experimental discharge lateral gap. An illustration of the experimental setup is presented in Figure 3. The high-speed camera VW9000, equipped with the VW600C lens, is configured for observing bubbles. This camera can achieve a maximum frame rate of 20,000 fps. To examine the morphological features of bubbles in various-sized regions, the long macro zoom unit VW—Z2 is employed as the focusing lens, with a maximum observation distance of 284.4 mm. The discharge current signal is acquired by a current transformer (MODEL 3972, Pearson ELECTRONICS). The discharge voltage signal is simultaneously captured by an oscilloscope (WAVESURFER 3034, TELEDYNE LECROY). This setup enables real-time monitoring of the discharge condition. User-defined discharge machining parameters and electrode feeding depth are inputted into a host computer equipped with LABVIEW software. It is worth noting that to minimize the impact of other factors on the bubble dynamics in electrical discharge machining, the nozzle’s flow rate is extremely low and is merely used to maintain a continuous inflow of working fluid into the machining gap.

### 2.2. Elucidation of the Interelectrode Discharge Condition Determined by Bubbles

**Figure 4:**
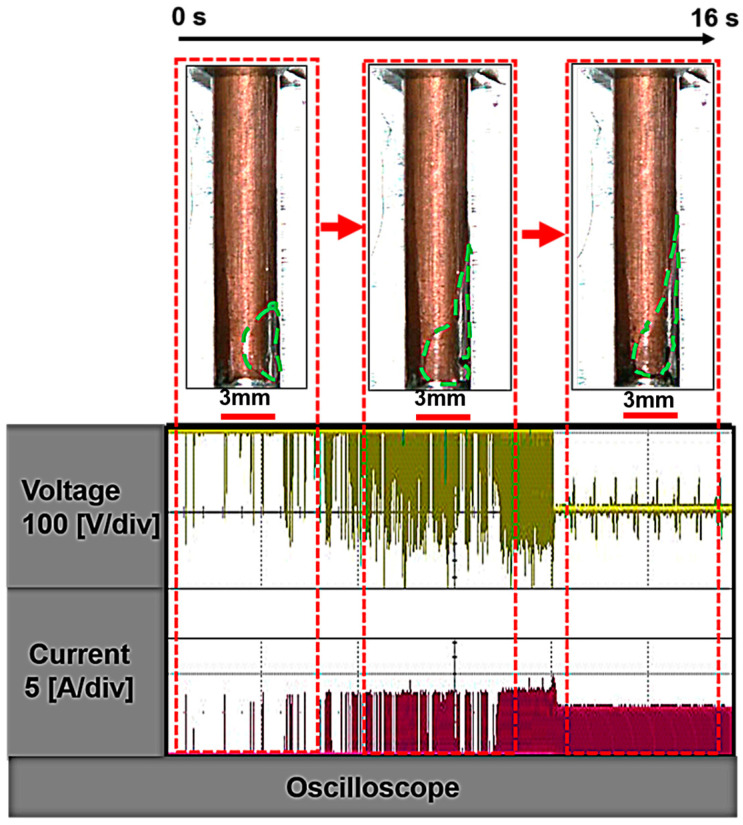
Currents and voltage waveforms in the high-aspect-ratio deep-hole EDM. Green dashed lines represent the bubble profile.

To investigate the impact of bubble behavior on the discharge conditions in the EDM process, we recorded gap current–voltage waveforms during EDM hole machining with an aspect ratio of 5, as depicted in Figure 4. In this experiment, the hole depth was set to 15 mm, and the machining time was limited to 16 s due to persistent short circuits occurring after this duration. As illustrated in Figure 4, a substantial number of bubbles emerged from the bottom discharge gap, coalescing to form a larger bubble that began to expand into the side gap as soon as the electrical discharge commenced. It is noteworthy that during this initial period, the voltage waveform showed no signs of short circuits. Instead, an open circuit state prevailed, accompanied by high voltage and a very weak gap current. As for the subsequent stages of machining, the bubble continued to enlarge and undergo deformation, transitioning from its initially spherical shape, which was near the wall. As indicated by the red dashed line, a typical spark discharge state was observed during this phase, characterized by discharge voltage fluctuations and breakdown delays. As the machining process progressed, the bubble shifted slightly upward, with its leading edge remaining attached to the electrode surface. However, at approximately 12 s to 16 s into the process, a short circuit occurred. During this time, bubbles occupied a significant portion of the space between the poles and the side gap as depicted on the right side of Figure 4. It can be inferred that issues related to bubble ejection contribute to unstable electrical discharge conditions, ultimately diminishing the efficiency of the material removal processes.

### 2.3. Depth-Dependent Bubble Behavior in EDM 

**Figure 5:**
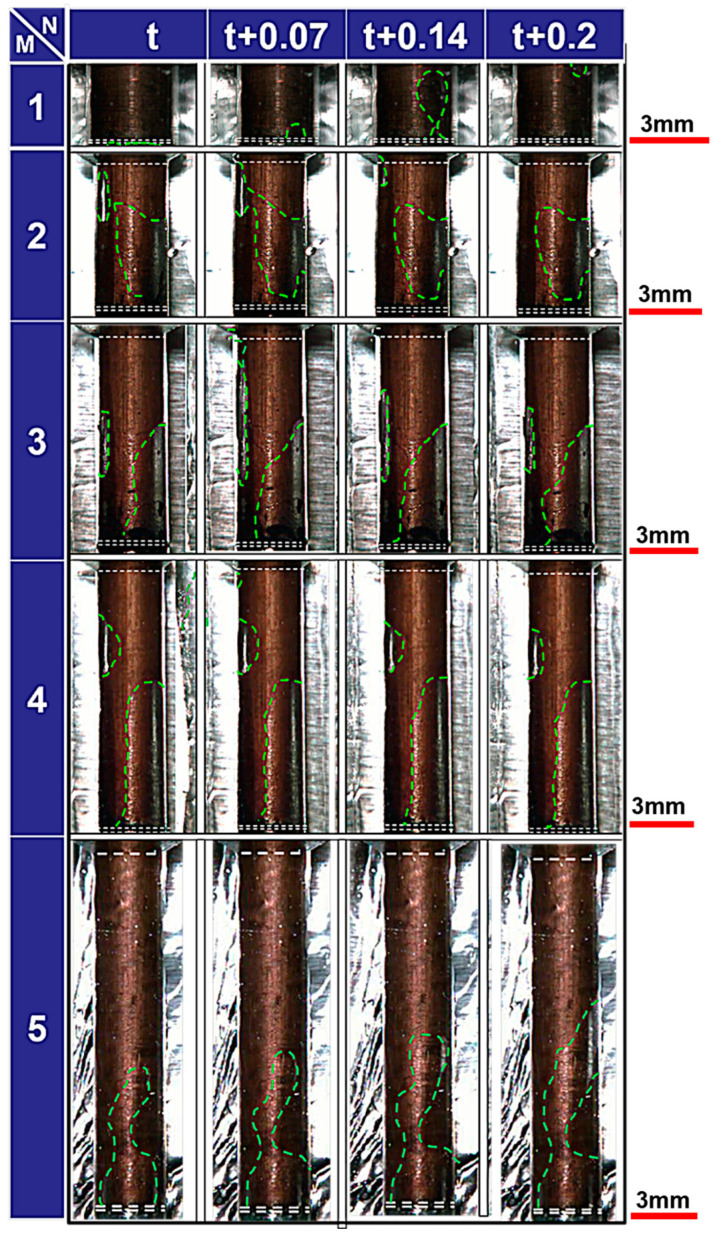
Images of bubbles in the gap captured at different feed depths and different times. The aspect ratio and time are denoted by M and N respectively. The bubble’s outer profile is indicated by green dashed lines.

The observational findings for the side gap in each aspect ratio are presented in Figure 5. The upper right corner of the figure shows the discharge of air bubbles with an aspect ratio of L/D = 1. Notably, there was no stagnation of bubbles between the poles and the side gap. It is essential to emphasize that machining debris was randomly distributed along the boundary between bubbles and that the liquid was carried by the movement of bubbles. However, regrettably, this phenomenon is not clearly depicted in the figure due to limited image resolution. Similarly, the discharge of bubbles is confirmed for L/D = 2 and L/D = 3, as shown in the upper left portion of the image. In subfigure (c) at t + 0.14 s, a bubble is discharged from the upper left for L/D = 4, but subsequent bubbles become stagnant between the poles and along the side. Notably, within the 20 s observation period using a high-speed camera, no bubble exclusion was observed for L/D = 5. This demonstrates that air bubbles persist in the side gap and do not progress further in the EDM of holes with higher aspect ratios.

These experiments focused on examining the growth and deformation of bubbles during the EDM process. As the aspect ratio of the hole increased, the portion occupied by bubbles also expanded. For instance, when machining a hole with a depth of only 3 mm at L/D = 1, the bubbles were promptly discharged from the side gap. However, at L/D = 5, where the machining hole reached a depth of 15 mm, the path for bubble discharge became considerably longer. Consequently, the bubbles were unable to remain within the side gap and rapidly exited. In such cases, the side gap was predominantly filled with air bubbles, and these bubbles tended to merge with nearby ones, forming larger bubbles. In deep-hole EDM, it is believed that machining debris, whose movement is constrained, can lead to short circuits and abnormal discharge. This phenomenon aligns well with the discharge conditions observed in deep hole EDM, as discussed in the preceding section.

## 3. Effect of Ultrasonic Vibration on the Bubble Behavior in USV-Assisted EDM

As outlined in the previous section, the performance of EDM is significantly affected by the concentration of debris and the percentage of bubble volume in the bottom gap. High-speed camera observations revealed that when the machining depth of the hole reached an aspect ratio of 5, substantial bubbles coalesced with neighboring smaller bubbles, resulting in the formation of a large bubble. This large bubble generated localized and surface stress. Undoubtedly, the issue of coalescing bubbles occupying a substantial portion of the interior space within the discharge gap leads to frequent instances of abnormal discharges. The introduction of ultrasonic vibration in deep EDM is anticipated to enhance machining speed and ameliorate the deteriorating discharge environment. Consequently, in the following section, we will examine how ultrasonic vibration responds to discharge gap fluid flow and influences bubble behavior.

### 3.1. Finite Element Simulation of the Ultrasonic Vibration System

**Figure 6:**
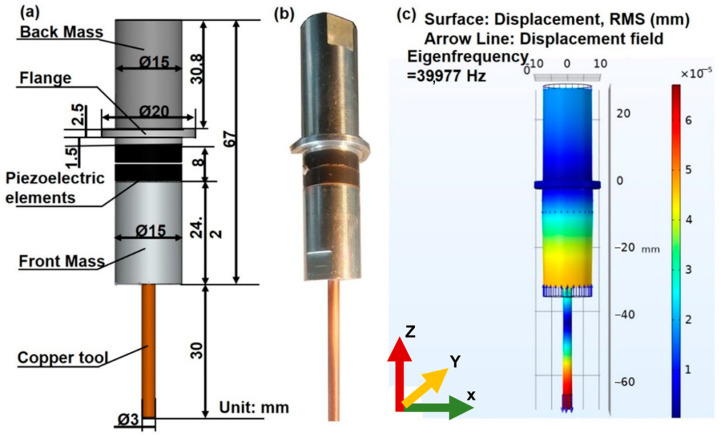
The results of modal analysis for the ultrasonic vibration system. (**a**) The geometry of the ultrasonic vibration system, (**b**) photograph, and (**c**) modal analysis with desired mode shape: the displacement amplitude and displacement field are indicated by the blue solid arrow.

Any study of ultrasonic vibration-assisted machining must necessarily proceed with a discussion of the system vibration characteristics. Mode analysis is typically employed to find the resonance frequencies, mode shapes, and the location of the nodal plane. In this work, we apply the COMSOL software to analyze the electrode vibration condition. As shown in Figure 6, the Langevin vibrator is adopted as the transducer which consists of a back mass, two piezoelectric rings, and a front mass. The back mass is made of AISI 4340 steel with a mass density of 7.85 g/cm^3^, a Young’s modulus of 205 GPa, and a Poisson’s ratio of 0.28. The material of the front mass is aluminum with a mass density of 2.70 g/cm^3^, a Young’s modulus of 70 GPa, and a Poisson’s ratio of 0.33. Two pieces of ring shaped piezoelectric ceramics are clamped in the middle of the transducer by thread fastening. Moreover, two piezoelectrical ceramics are applied with two voltage excitations of reverse phases to accomplish the push–pull excitation, hence realizing a whole vibration longitudinally. The flange is used to fix the transducer and the whole vibration system by applying displacement constraints of the clamping mechanism.

The geometry dimensions of the transducer and electrode are shown in Figure 6. The 2D axis-symmetric model of the vibration system was developed for both modal and frequency domain study. To ensure the accuracy of computed modal results, this study employed COMSOL Multiphysics to conduct a mesh convergence study. The physical settings were configured for Solid Mechanics, and the mesh refinement process was systematically performed during the grid partitioning stage. Each refined mesh from this process was treated as a new analysis entity for modal analysis. A comparative evaluation of the first 10 order longitudinal vibrational natural frequencies was carried out at each refinement step. Ultimately, the converged mesh was employed for the final modal analysis. The ultimate longitudinal natural mode was obtained, as shown in Figure 6. The result of modal analysis showed that the longitudinal vibration eigenfrequency was 39,977 Hz and that the flange was in the position of the nodal plane. Furthermore, it can be recognized from the displacement field in Figure 6 that the longitudinal deformation of the electrode tip reached a maximum under the eigenfrequency of 39,977 Hz.

**Figure 7:**
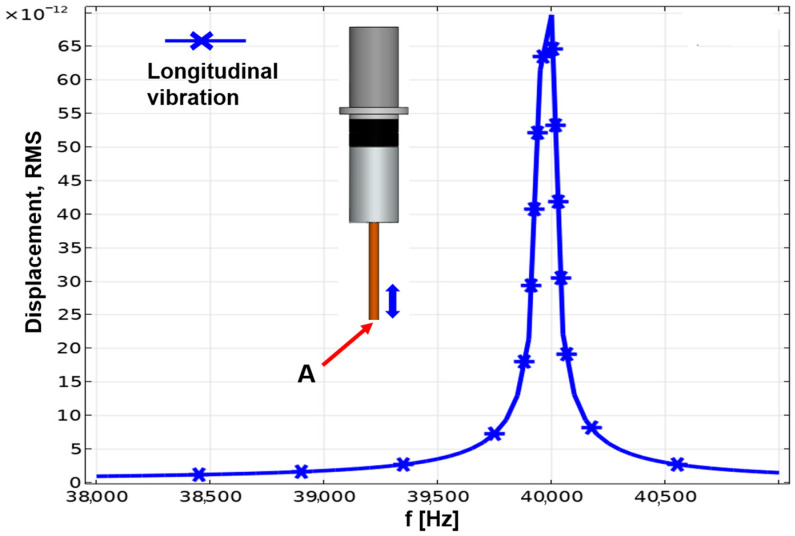
Relation between the amplitude of tip A and vibration frequency according to harmonic analysis.

To analyze the response of the assumed linear model under harmonic excitation at various frequencies, a frequency domain analysis was conducted, as detailed in [17]. The harmonic disturbance was applied to the vibration system as an excitation, and a frequency sweep calculation was performed around 39,977 Hz. The results of the frequency domain studies are typically presented in the form of a transfer function, and Figure 7 illustrates the displacement in terms of root-mean-square versus frequency. From the figure, it is evident that the resonance frequency of the longitudinal vibration system was approximately 40 kHz. Therefore, the frequency of the function generator’s AC voltage was set to 40 kHz. The function generator and amplifier composed the ultrasonic power supply, and the Langevin-type vibrator was used to generate the ultrasonic vibration, which was then sent to the electrode. A high-resolution laser displacement sensor fabricated by KEYENCE company was utilized to measure the electrode displacement. The electrode tip amplitude measured in this study was 2 µm. The subsequent fluid simulation section includes a selection of 2 µm as the amplitude for the moving wall.

### 3.2. CFD Simulation of Interelectrode Fluid Flow with Ultrasonic Vibration

It was found that applied ultrasonic vibration to the electrode strongly influenced the EDM performance. Since a dielectric undergoes a periodic flow velocity and pressure distribution change due to fluid flow subjected to ultrasonic vibration, it is of interest to investigate the electrode boundary and dielectric flow interactions. The velocity and pressure of discharge gap fluid flow are presented in this section under the given ultrasonic vibration parameters. Fluent software was adopted in this simulation due to its’ offering of a variety of turbulence models, ranging from standard models such as k-ε and k-ω to more advanced models.

#### 3.2.1. Principle of the Ultrasonic Flow Field

For the convenience of analyzing the interelectrode gap flow field, reducing the difficulty in analyzing the interelectrode gap flow field, and better understanding the motion state of the interelectrode gap flow field, the following assumptions are made for the working fluid between the electrodes: the working fluid in the interelectrode gap flow field is considered a continuous and incompressible medium, and the fluid flow is assumed to be steady. Additionally, thermal transfer is not considered during the processing [18]. Based on these assumptions, the fluid and bubbles are regarded as a single liquid phase.

#### 3.2.2. Selection of the Simulation Model

For the fully developed turbulence and solving flows in the core region, it is convenient to use a high Reynolds number model. We employ the standard *k*-epsilon (*k-ε*) turbulence model in this work. The governing equation is given by [19,20] for turbulent kinetic energy ***k***:

(1) $$\frac{\partial (\rho k)}{\partial t}+\frac{\partial \left(\rho ku_{i}\right)}{\partial x_{i}}=\frac{\partial }{\partial x_{j}}\left[\frac{\mu_{t}}{\sigma_{k}}\frac{\partial k}{\partial x_{j}}\right]+2\mu_{t}E_{ij}E_{ij}-\rho \varepsilon$$

and for dissipation ε:

(2) $$\frac{\partial (\rho \varepsilon )}{\partial t}+\frac{\partial \left(\rho \varepsilon u_{i}\right)}{\partial x_{i}}=\frac{\partial }{\partial x_{j}}\left[\frac{\mu_{t}}{\sigma_{\varepsilon }}\frac{\partial \varepsilon }{\partial x_{j}}\right]+C_{1\varepsilon }\frac{\varepsilon }{k}2\mu_{t}E_{ij}E_{ij}-C_{2\varepsilon }\rho \frac{\varepsilon^{2}}{k}$$

where $u_{i}$ represents the velocity component in the corresponding direction, $E_{ij}$ represents a component of the rate of deformation, and $\mu_{t}$ represents eddy viscosity.

(3) $$\mu_{t}=\rho C_{\mu }\frac{k^{2}}{\varepsilon }$$

Several following empirical constants are defaulted to as follows:

(4) $$C_{1\varepsilon }=1.44;\;\sigma_{k}=1.00;\;C_{2\varepsilon }=1.92;\;\sigma_{\varepsilon }=1.30;\;C_{\mu }=0.09$$

The standard wall function is activated in this model. Fluent can apply simplified models to represent the turbulence near the walls, allowing for larger mesh sizes in the bulk flow region while maintaining accuracy in the near-wall region. This helps in reducing the computational cost of the simulation. Fluent solution methods are described as follows. The simulation uses the SIMPLE (semi-implicit method for pressure-linked equations) algorithm for iterative pressure–velocity coupling in solving fluid flow equations. It employs a least squares cell-based approach for equation discretization, applying second-order numerical schemes for both spatial discretization and the advection term. Additionally, a first-order implicit scheme is used for time integration in governing equations.

#### 3.2.3. Dynamic Mesh Establishing and Parameter Settings for Gap Flow Field Calculation

**Figure 8:**
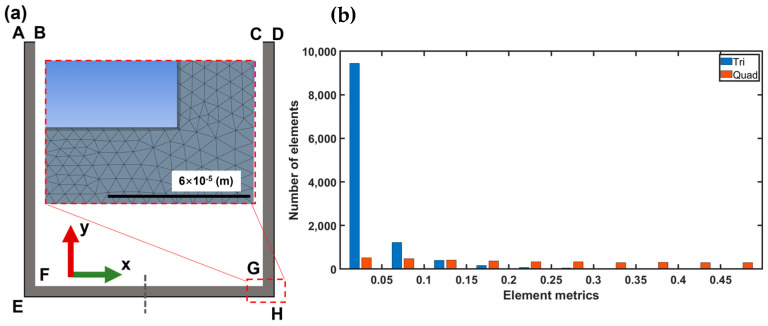
(**a**) Two-dimensional profile of interelectrode fluid model: AB and CD are pressure outlets with the same length of 0.05 mm. AE, DH, and EH are the wall with a length of 3.05 mm, 3.05 mm, and 3.1 mm, respectively. FG, BF, and GC represent the electrode tip immersed in the kerosene with the same length of 3 mm. (**b**) Mesh metrics.

A two-dimensional fluid flow geometry model was established according to the USV- assisted EDM schematic (see Figure 8). In this model, FG, BF, and GC are the side surfaces of the electrode. AE, DH, and EH represent workpiece surfaces that are set up to fixed walls. Ultrasonic vibration of the electrode is set by dynamic mesh as indicated in moving wall FG. Moving wall moving velocity is given by and compiled with UDF (user-defined function) in Fluent solver:

(5) $$v=A\cdot 2\pi f\cdot \cos\left(2\pi ft\right)$$

where *A* is the ultrasonic vibration amplitude and equal to 2 μm, which is measured with the laser displacement sensor in our previous investigation, and the resonance frequency denoted by *f* is 40 KHz. The gauge pressure at the outlet, represented by AB and CD, is set to zero by default. The mesh file is generated by a default mesh generation tool in ANSYS Workbench. To enhance grid convergence in this study, the following configurations were implemented. Adaptive sizing was employed, with the grid quality set to the highest level of smoothness. To simulate the behavior of fluid more accurately within the boundary layer and improve simulation precision, precise wall boundary conditions were provided using “inflation”. Specifically, a four-layer refined mesh was applied to the corresponding walls. As depicted in the Figure 8b, the skewness of the grid, comprised of both quadrilaterals and triangles, was consistently below 0.5. This strongly indicates a high-quality mesh, conducive to the stability and accuracy of the simulation.

#### 3.2.4. Analysis of the Simulated Flow Field

**Figure 9:**
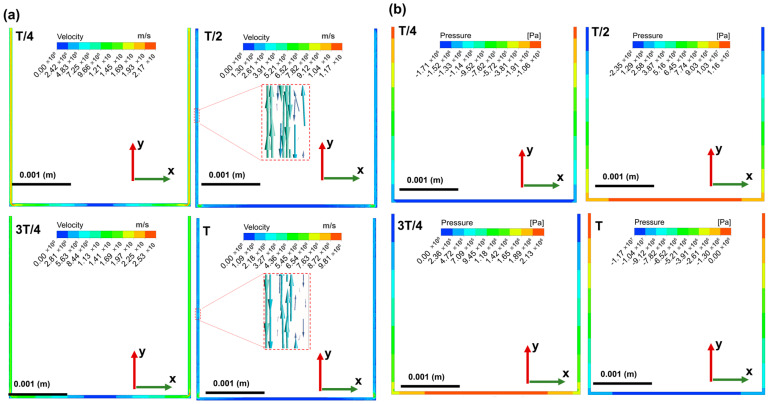
(**a**) Velocity cloud charts in the interelectrode flow field of the X–Y plane. (**b**) Pressure changes cloud charts in the interelectrode flow field of the X–Y plane.

Four time points (T/4, T/2, 3T/4, T) in one ultrasonic vibration cycle are selected in the interelectrode fluid flow model. In Figure 9a, we present the flow field velocity at four different time points. The longitudinal ultrasonic vibration of the electrode causes the flow velocity of the discharge gap and lateral gap machining fluid to change repeatedly. At any given time, the fluid flow velocity at the center of the bottom discharge gap is the lowest, while the flow velocity near the electrode corner is always higher. Ultrasonic vibration causes large fluctuations in the flow layer at the corner position, the liquid always forms a vortex around the corner. The center of the bottom discharge gap where the local velocity of the fluid is almost zero is called the stagnation zone [21]. At this zone, the flow completely stops, thereby converting its kinetic energy into pressure; this is shown in Figure 9b, in which the pressure value reaches the maximum at the center of the bottom discharge gap. As demonstrated in Figure 9a, assuming the initial position of the electrode is at the lowest end, when time proceeds from 0 to T/4, the electrode moves upward from the workpiece, and thus the discharge gap increases. The dielectric fluid begins to move from the lateral side gap to the bottom side. At point T/2, the electrode velocity is zero, and the discharge gap reaches the maximum. As seen from the localized magnification of the velocity vector plot shown in the Figure 9a, at this time point, within the side discharge gap, the fluid velocity near the wall is smaller than the fluid velocity farther from the wall, and they are in opposite directions. This could be attributed to the viscous effects causing the fluid velocity away from the wall to exceed that of the near-wall fluid within the boundary layer. This situation is also observed at time T. When the time proceeds from T/2 to 3T/4, the electrode moves from the highest point to the vibration center. At the 3T/4 time point, the velocity of moving wall has the maximum downward velocity, and the overall velocity of the flow field increases significantly, thus potentially altering the flow and forces acting upon the debris and bubbles [22]. Note that at point 3T/4, the fluid velocity of the lateral gap reaches a peak at about 15.2 m/s. When time changes from 3T/4 to T, the wall continuously descends until reaching the vibration center. At this juncture, the fluid at the bottom of the gap is squeezed toward the side gaps. As the wall reaches the lowest position, the velocity decreases to zero.

The pressure change cloud charts during a single-vibration cycle are illustrated in Figure 9b. One of the consequences of the introducing ultrasonic vibration to EDM is the repeated pressure change of the discharge gap flow field. The obvious feature of the flow field is that pressure changes from negative values to positive values and then reverses back to negative values. As shown in Figure 9b, from T/4 to T/2, the wall moves upward along the y-axis, departing from the vibration center. When reaching the T/2 time point, the wall velocity is zero. However, at this moment, the pressure at the side gap reaches its maximum value of 1.16 × 10^7^ Pa. Under this condition, it can be concluded that vapor bubbles within the fluid are extremely prone to rupture in these high-pressure areas, generating shock waves and thus improving the debris exclusion. At time T, the distance between the workpiece and the electrode is minimized, and at this moment, the pressure in the discharge gap reaches its minimum value, approximately −1.17 × 10^7^ Pa. Furthermore, the flow pressure distribution is symmetric at the X–Y plane. The periodic pressure variations may induce cavitation effects in the working fluid, leading to the formation of bubbles in low-pressure regions. The collapse of these bubbles generates shock waves and localized high temperatures. These effects contribute to improving material removal efficiency in electrical discharge machining, facilitating easier erosion and removal of material from the workpiece surface. Unfortunately, in this study, coupled simulations of ultrasonic cavitation were not conducted. Future work will further explore the influence of ultrasonic cavitation on the performance of electrical discharge machining.

## 4. Bubble Behaviors in the High-Aspect-Ratio Hole Ultrasonic Vibration-Assisted EDM 

**Figure 10:**
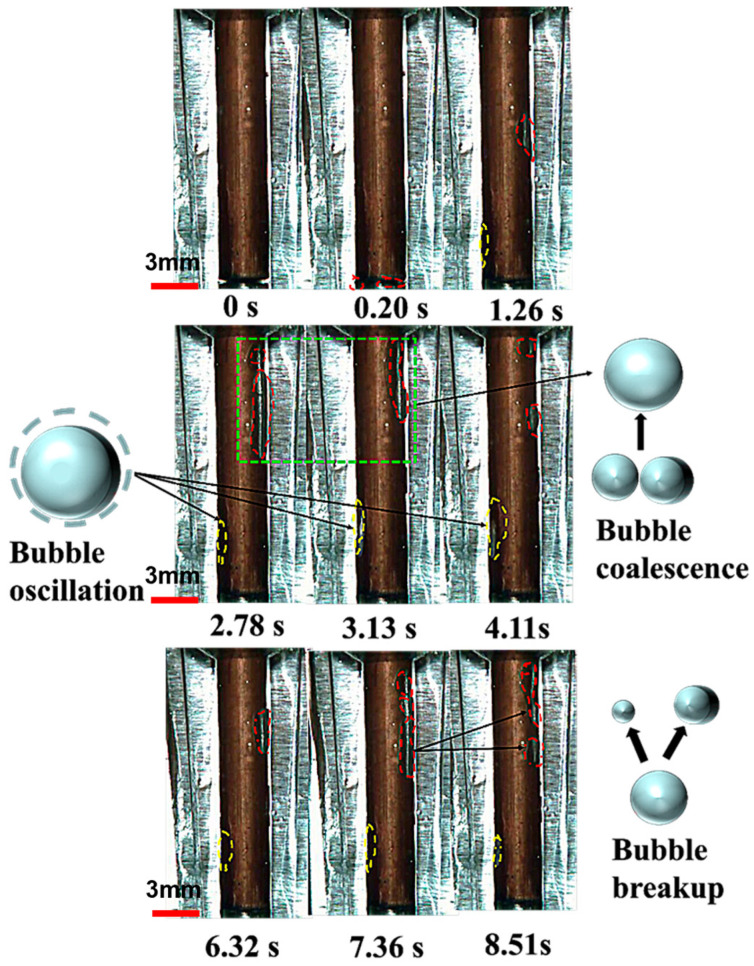
Photographs taken at intervals during USV-assisted EDM reveal bubble behavior on the electrode under 40 kHz resonance frequency and a longitudinal amplitude of 2 μm.

In this section, our focus is on examining the formation, deformation, and movement of bubbles under ultrasonic vibration conditions in an EDM hole with an aspect ratio of 5. Initially, as the electrode approaches the workpiece to the threshold breakdown distance, the gap area experiences breakdown. Following this, an arc channel forms between the electrode and the workpiece, leading to the formation and expansion of bubbles around the arc plasma. This is a result of the significant temperature generated by high-speed collisions among charged particles within the plasma arc column. As illustrated in Figure 10, by 0.20 s, the gap between the electrode and workpiece is primarily occupied by bubbles. Unfortunately, the rapid expansion of bubbles within the gap occurred so swiftly in our study that we were unable to capture this moment in a photograph. Simultaneously, bubbles emerge from the bottom discharge gap and rise in the liquid. At 1.26 s, two types of bubbles stagnate in the side gap. Bubbles consistently located on the left side of the gap are highlighted by a yellow dashed line, while those on the right side are marked with a red dashed line. From 2.78 s to 4.11 s, as shown in Figure 10, the bubble on the right side moves upward along the electrode. This dynamic behavior of the bubble differs from that observed in EDM without USV, as described in Section 2.3. However, the bubble on the left side remains stagnant and oscillating. Although not depicted in the figures, this behavior can be observed in the video provided. The differing bubble behavior may be attributed to the asymmetry in the left and right gaps during the workpiece insertion process. Figure 10 highlights an important observation: bubbles coalesce to form a larger bubble, which is then expelled from the outlet at 3.13 s. This occurs because the rise velocity of the bubble decreases, leading to bubble accumulation. Additionally, the buoyancy force diminishes as the bubble ascends.

However, this is not always the case in EDM without USV. When compared with previous bubble observation results, in the EDM hole with an aspect ratio of 5, the bubble stagnates in the lower half of the side gap and cannot continue moving. Instead, with the assistance of USV, at 7.36 s, we observe significant deformation and even rupture of the larger bubble. This may occur because the ultrasonic vibration of the electrode enhances the vortex flow and aids in the swift removal of bubbles; meanwhile, the collapse phase generates shock waves and localized high temperatures, promoting bubble rupture and expulsion from the machining area [23,24].

## 5. Conclusions

To investigate the influence of ultrasonic vibration on the bubble behavior in EDM in this study, we machined holes of varying depths on a transparent acrylic tank to simulate the discharge gap. Observations were conducted using a high-speed camera, and CFD simulations were performed. The most important results are as follows:

(1) As machining depth increases, it increasingly challenging for bubbles to exit the interelectrode gap, leading to the predominant occupation of the side gap by bubbles.
(2) Observation results indicate that debris freely distributes along the liquid–gas boundary, with its removal primarily dependent on the driving force exerted by bubbles.
(3) CFD results indicate that periodic variations in flow pressure and velocity induce turbulence, potentially positively impacting the exclusion of bubbles and discharge of debris in deep-hole EDM.
(4) In USV-assisted EDM of holes with an aspect ratio of 5, bubbles consistently fragment and coalesce toward the hole outlet. In contrast, without ultrasonic vibration in EDM for holes with smaller aspect ratios, bubbles tend to stagnate and oscillate within the interelectrode gap without further progression.
(5) As the depth-to-diameter ratio of the machining hole exceeds 5, the expulsion of machining bubbles and debris solely relying on the pumping and cavitation effects induced by the ultrasonic vibration at the electrode tip are insufficient. Therefore, future research will emphasize the investigation of multimodal vibration of the electrode to enhance the performance of USV-assisted EDM.

## Data Availability

Data are contained within this article.
